# Rate-Distortion-Based Stego: A Large-Capacity Secure Steganography Scheme for Hiding Digital Images

**DOI:** 10.3390/e24070982

**Published:** 2022-07-15

**Authors:** Yi-Lun Pan, Ja-Ling Wu

**Affiliations:** 1 Department of Computer Science and Information Engineering, National Taiwan University, Taipei 10617, Taiwan; d06922016@csie.ntu.edu.tw; 2 National Center for High-Performance Computing, Hsinchu 30076, Taiwan; 3 Graduate Institute of Networking and Multimedia, National Taiwan University, Taipei 10617, Taiwan

**Keywords:** image steganography, information hiding, rate-distortion, mutual information, generative adversarial network

## Abstract

Steganography is one of the most crucial methods for information hiding, which embeds secret data on an ordinary file or a cover message for avoiding detection. We designed a novel rate-distortion-based large-capacity secure steganographic system, called rate-distortion-based Stego (RD-Stego), to effectively solve the above requirement. The considered effectiveness of our system design includes embedding capacity, adaptability to chosen cover attacks, and the stability of the trained model. The proposed stego scheme can hide multiple three-channel color images and QR codes within another three-channel color image with low visual distortion. Empirically, with a certain degree of robustness against the chosen cover attack, we state that the system offers up to 192+ bits-per-pixel (bpp) embedding of a payload and leaks no secret-related information. Moreover, to provide theoretical foundations for our cost function design, a mutual information-based explanation of the choices of regulation processes is herein included. Finally, we justify our system’s claimed advantages through a series of experiments with publicly available benchmark datasets.

## 1. Introduction

Information hiding can imperceptibly transfer secret information into chosen cover media [1]. It can ensure the origins of data and behave as a second channel for data transmission. Steganography is the art of covering or hiding extra data inside a chosen cover message, e.g., an image. The term itself dates back to the 15th century; in a typical scenario, the sender hides a secret message inside a cover image and transmits it to the receiver, who recovers the message. Even if eavesdroppers monitor or intercept the communication in-between, no one besides the sender and receiver should detect the presence of the hidden message. Compared to cryptography, steganography has the advantage that non-target intermediaries will not suspect the existence of secret information itself. The media embedded within extra messages is called the stego media, and the media used to host the embedded messages are called the cover media. Attackers use steganalysis techniques to prevent the successful transmission of secret information. To conduct steganography is challenging because embedding extra messages can alter the cover’s appearance and underlying statistical distribution.

The first common challenge in designing a steganography scheme is how to enlarge the amount of transmittable payload, named the scheme’s capacity. Steganography capacity is usually measured in *bits-per-pixel* (bpp). The longer the embedded message, the larger the bpp and the more altered the cover. Suppose the visual appearance of the hidden-message embedded image (denoted as the stego-image) does not appear close to that of the cover images. In that case, non-photo-realistic issues may result in the associated synthesis-based applications, such as the anchor face generation application in the metaverse. Existing image steganography approaches are only practical for embedding a relatively low payload of around 0.4 bits per pixel [2]. With vigorous developments in generative adversarial networks (GANs), many works have applied GAN-based approaches to steganographic design methods [3,4,5], which saw a boom in image steganography’s applicability. Reference [6] is the first article that attempts to address the application of GAN in image steganography with acceptable performance. Afterwards, with the help of GAN, Zhang et al. proposed the SteganoGAN [7], which achieves the embedding capacity with a payload of 4.4 bits per pixel. In 2020, Fu et al. improved the work of SteganoGAN; they proposed the HIGAN [8], which can handle a 24-bit-sized payload. Investigating the possibility of further increasing information capacity is one of the to-be-conquered challenges of this study.

Furthermore, to enlarge the information embedding capacity to higher than 192+ bpp, inspired by the authors of [9], we leverage the rate-distortion loss functions to ensure the visibility of the cover image and enhance the compressibility of the embedding image. In other words, our primary goal is to optimize the visual quality of the stego-image and hide as much secret-related information as possible at the same time.

The second challenge of steganography is its poor robustness against the chosen cover attacks [6]. When an attacker knows both the stego and the cover images, conducting a simple pixel differencing operation may leak secretly-related information. Although the recent work proposed by Lu et al. [10] can hide multiple secret images, low system security against simple pixel-differencing operations is still the main weak point of the approach, i.e., the confidential information will be exposed. In contrast, our proposed multiple-secret-image embedding scheme, besides enlarging the capacity, will also significantly improve the system’s security.

The third challenge concerns the stability of the trained model. Most of the related works developed a supervised cover synthesis steganography, as addressed in [11], to face the model’s training stability issues.

As for the state-of-the-art in the field of NN-based steganography published in the past three years, we recommend the following five highly related works: Duan et al. [12], SteganoGAN [7], HIGAN [8], SteganoCNN [13], and ISN [10]. Among them, [7,8,13] are limited in their model capabilities and can only process a singular secret image or text information. Nevertheless, it is worth noting that the quality of the images processed in [12] is superior. Inspired by [12], we also tried to make the quality of the generated stegos and the reconstructed images as good as possible. SteganoCNN increased the embedding payload capacity to 47.92 bpp, while ISN considered how to handle multiple secret images hidden. Increasing the embedding capacity and relatively high computational complexity are still weaknesses of these proposals. Our work reduces the computational complexity from the perspective of network architecture. In summary, compared with the works mentioned above, our approach enlarged the payload capacity, enhanced the computational stability, and increased the computational efficiency simultaneously.

Besides, most of the above studies did not provide theoretical information-based analyses of their work, which might bring further insights for comprehending the approaches’ physical meaning. To respond to this concern, we not only propose the RD-Stego system but also provide an informational-theoretic explanation of the design of the adopted cost functions. We take Shannon’s mutual information (MI) into the construction of the RD-Stego system’s cost functions, including (a) visual acceptability—in maximizing the MI lower bound of the difference between the cover and the stego-images, which is equivalent to maximizing the acceptable perception range between them. (b) Recovery fidelity—maximizing the MI lower bound between the embedded secret and the reconstructed secret, which is equivalent to maximizing the retrieval fidelity related to the secret messages.

Inspired by HIGAN [8,9], this work designs a novel rate-distortion-based, large-capacity, secure, semi-supervised cover synthesis steganographic system, i.e., the RD-Stego. It can hide multiple full-color secret images with N * N * (RGB) (i.e., 256 * 256 * 3) pixels and QR-coded image pixels into another N * N * (RGB) cover image of the same size with low perceptible distortion to the cover. The proposed RD-Stego provides relatively large information capacity and can resist chosen cover attacks compared to previous works. Notice that because RD-Stego can smooth the discontinuity of the gradient calculation during training (we will address it in later sections), the RD-Stego trained model is relatively stable.

The proposed RD-Stego is a semi-supervised synthesis steganography algorithm that establishes a mapping between the class labels of the generated images and the secret information to be embedded automatically. Thus, there is no human intervention needed during network training. In addition, the advantages of such a design can also prove that the visual acceptability and recovery radiality cost functions can guide the learning of RD-Stego for more stable training in the subsequent theoretical information-theoretic analysis. Moreover, coupled with the design of the rate-distortion loss function, the RD-Stego can do elementwise addition, channel by channel, on each secret image vertically, allowing the encoder to perform encoding training more efficiently.

The contributions of this work can be summarized as follows:

- Providing an informational-theoretic-based high capacity steganographic algorithm to hide multiple security-sensitive messages, such as multiple RGB images and QR-coded images;
- Using rate-distortion theory to ensure better fidelity of the stego-image and increase the compressibility of the embedded secret images (the information embedding capacity is higher than those within the existing competing works, with better or similar PSNR ratios);
- Enhancing the system’s security with appropriate machine learning techniques. The proposed RD-Stego can survive the chosen cover attacks, which is another strong point compared to previous works;
- Deriving maximized MI lower bounds for the cover vs. the stego and the embedded secret vs. the reconstructed secret during network training, which provides reasonable regulations for the training process and enhances the stability of the trained model;
- Justifying the claimed ability to embed and reconstruct many payloads, such as multiple full-color images and QR-coded images, through a series of concrete experiments.

We summarize the advantages and the limitations of the proposed RD-Stego compared with the related works in Table 1. We have added the “information-theoretic analyses” as one of the comparison items to emphasize the specific contribution of the proposed RD-Stego.

To verify our claims, we use the following datasets—FaceScrub [14], CASIA-WebFace [15], and CelebA-HQ/CelebA [16] to train the proposed model and use ImageNet [17] to evaluate and test for cross-domain performance. Experimental results show that the proposed approach can generate photo-realistic stego-images without sacrificing the embedded information capacity compared with all related methods.

## 2. Related Works

This section briefly reviews the recent progress in steganography based on GANs and focuses specifically on the limitations in the embedding capacity and the ability to resist attacks.

### 2.1. Steganography Based on GANs

With the great help of GAN, several researchers found that GAN-based steganography can solve the problem of non-photo-realistic appearance in cover synthesis. Abadi et al. [18] first applied this idea to steganography’s cover synthesis and added an adversarial network to their algorithm. Zhu et al. [19] proposed an encoder-decoder network architecture to deal with the embedding and extraction of secret information. The shortcomings of [18,19] are the adopted loss functions, which complicate the system design and make the training process unstable. Zhang et al. [7] significantly improved the loss function design and presented an end-to-end GAN-based steganographic model. They used adversarial training to solve the steganography task and regarded message embedding and extraction as encoding and decoding problems. Tancik et al. [20] achieved robust decoding even under “physical transmission” by adding a set of differential image corruptions between the encoder and decoder that successfully approximate the space of distortions. However, in the above three articles, the steganographic images generated by the neural network are highly correlated with the original cover.

Hu et al. [21] tried to accomplish the cover synthesis of steganography in an unsupervised manner. The key idea is finding a map from the noise to message and hiding messages into noises. A special extractor is then trained to extract messages from the noise. However, the high implementation cost of the latter training handicaps its value in practical usage. In response to unsupervised cover synthesis steganography being hard to use in practice, subsequent works redirect themselves toward the semi-supervised counterparts instead. Inspired by ACGAN, Liu et al. [22] proposed establishing a mapping relationship between the class label and noise first and then generating stego-images. Our proposed RD-Stego model leverages the advantages of semi-supervised cover synthesis steganography algorithms. In our work, the encoder network comprises a convolution layer and the residual block. As a result, the generated steganography image has much lower distortion and closer distribution to the original carrier image. Moreover, our work can smooth the discontinuity in gradient calculation during training. Such a smoothing gradient calculation characteristic provides reasonable training stability and conforms to steganographic basic conditions (BSC) [23,24].

- $S_{stego}=Emb(c|C,\;m,k_{emb})$, where *Emb(.)* denotes a data-embedding method based on a specific carrier *c* or a set of carriers *C*. The sender needs to design a scheme to construct stego media $S_{stego}$ with an embedding key *k_emb_*.
- $m^{\prime }=Ext\left(S_{stego},k_{ext}\right)$, where *Ext(.)* denotes a message extraction operation, which needs the inputs $S_{stego}$ and the extraction key *k_ext_*. The receiver can recover a secret message $m^{\prime }$ by using *k_ext_* and the message extraction operation.
- $D_{distinguishability}\left(C_{cover},S_{stego}\right)\leq \varepsilon$, where $C_{cover}$ and $S_{stego}$ represent the cover set and the stego set, respectively, and ε stands for a quantifiable level of security for indistinguishability, the so-called ε-security.

### 2.2. The Limitations of the Current Steganography Works

At present, the most apparent limitations of GAN-based steganography algorithms are their low embedding capacity and low robustness against the chosen cover attacks. As for the embedding capacity, Baluja [6] presented an encoder–decoder network and tried to increase the amount of information it carried [6], successfully embedding a color image into another color image of the same size, yet the resulting stego-image may expose confidential information. Rehman et al. [25] tried to hide a gray-level picture into a color picture of the same size, but severe color distortion was observed in the resultant stego-image. Zhang et al. [26] proposed the ISGAN process, which hides a grayscale image into the Y channel of a color cover image and improves the security of the model through adversarial training between the encoder–decoder and steganalysis networks.

Zhang et al. [26] inspired us to use another channel to aggregate the information that needs to be protected. Besides traditional RGB color channels, we use an extra channel for hiding QR code/text information in our work. In this way, we can use the SteganoGAN [7] to hide the color, grey-scale, and binary data in a hosted picture and enlarge the information capacity contained in the stego-image. In doing so, SteganoGAN achieves 4.4 bits-per-pixel embedding capacity; this is still not good enough. Fu et al. [8] enlarged the payload of [7] in 2020. Whether it is possible to continue to increase the embedding capacity is the main target of this work. The lesson learned from [8] tells us that using other channels to handle non-color information, such as QR-coded messages, seems to be a good choice. In other words, if the designed RD-Stego can rebuild QR-coded messages perfectly, we will completely solve the embedding capacity issue.

Deep Steganography [6], proposed by Baluja, faces the problem of chosen cover attacks, especially when attackers have both the stego and cover images. The attackers can magnify the difference between the stego and the cover images and easily extract secret-related information. This shortage comes from the Deep Steganography method inputting both the cover and the secret images into its pre-trained model and then connecting them back into GAN in series. Therefore, an attacker can choose a specific cover image as input and subtract it from the associated stego-image to find their difference. To deal with this issue, Tang et al. [27] proposed an adversarial embedding scheme based on CNN-ADV-EMB architecture to resist the above-mentioned chosen cover attack. Unfortunately, this type of method is of a security concern. Instead of directly concatenating the cover and the stego-images, the proposed RD-Stego uses element-wise additions to perform perturbation, significantly enhancing system security. In 2021, although the method proposed by Lu et al. [10] can hide multiple secret images, the main weakness of the method is also apparent in terms of security, which requires a simple pixel-differencing operation for the secret information to be exposed. On the contrary, our proposed method also significantly improves security, especially for this problem.

## 3. The Proposed Approach

This section presents the proposed RD-Stego method in detail from the perspective of the following three aspects: (1) the network architecture—encoder and decoder framework, (2) the disentangle efficacy of the designed rate-distortion loss functions, and (3) the information-theoretic based analyses—cost functions.

### 3.1. The RD-Stego Network Architecture

**Figure 1:**
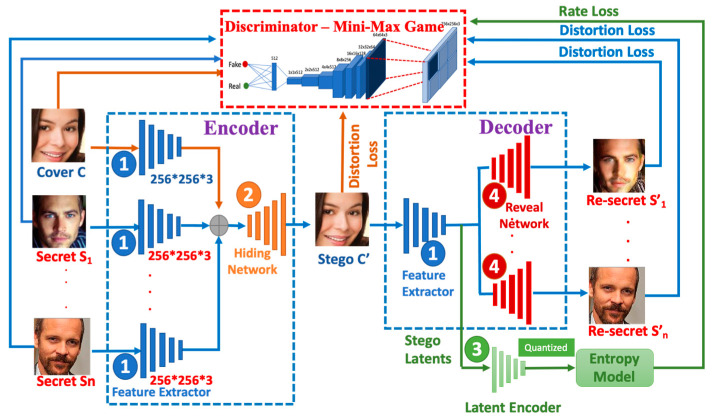
The architecture of the RD-Stego system.

Our RD-Stego network incorporates the encoder–decoder framework and the information maximization technique [28] to build a semi-supervised cover synthesis steganography system. The most important part is to emerge the rate-distortion idea of compression theory into the entire network architecture for enlarging the hiding capability, as shown in Figure 1. It consists of four networks, including:

1. An encoder uses a three-channel color cover image, multiple three-channel color secret images, and even a QR code as inputs to generate a stego-image;
2. A decoder takes the stego-image as the input and reconstructs the secret-related messages and the QR-coded messages as well;
3. A latent encoder takes the stego latents as the input and quantizes these stego latents to the nearest integer. Then, the entropy model proceeds to calculate the entropy between the stego latents and quantized stego latents;
4. A Discriminator uses PatchGAN-D [29] to judge whether the cover and the stego-images, the secret and the reconstructed secret photos, or the embedded QR-coded and the reconstructed QR-coded messages are similar.

As Figure 1 shows, the Encoder of the RD-Stego system consists of two subnetworks. One is the Feature Extractor (the subnetwork labeled as “1” and symbolized by blue rectangular blocks), and the other is the Hiding Network (the subnetwork labeled as “2” and symbolized by orange rectangular blocks). Feature Extractor is mainly responsible for processing input images, including cover images (C) and multiple secret images ($s_{1},s_{2},\ldots ,s_{n}$). In practice, dealing with a three-channel color image is more complicated than non-color information. Since we want RD-Stego to be capable of embedding more generic messages, the Feature Extractor is designed to be able to handle three-channel color and non-color images simultaneously. Our Feature Extractor puts the non-color images (e.g., QR-code or text information) on the blue channel. For processing, the proposed scheme regards a non-color image as a three-channel color image but pads zero values on the red and green channels. Next, the Feature Extractor performs elementwise addition vertically on all input images. It then feeds the results into the Hiding Network, which is in charge of generating the stego-image (C’), so the entire calculation work of the Encoder can be automatically executed.

Our RD-Stego’s Decoder consists of three subnetworks, including the Feature Extractor (also labeled as “1” and symbolized by blue rectangular blocks), the Latent Encoder (the subnetwork labeled as “3” and symbolized by green rectangular blocks), and the Reveal Network (the subnetwork labeled as “4” and symbolized by red rectangular blocks). On the one hand, the Feature Extractor extracts the secret image’s features from the stego-image (C’) and feeds the result into the Reveal Network for subsequent processing of the reconstructed secret images. On the other hand, in the meanwhile, the Feature Extractor also generates the Stego Latents and inputs them to the Latent Encoder. Then, the Latent Encoder is in charge of quantizing the latent codes and calculating the cross-entropy via the green-colored entropy model.

Our whole model behaves as a minimax game, and the goal is to let the encoder learn distributions $P_{En}\left(x\right)$ and $P_{De}\left(x\right)$ that match the hidden data distribution $P_{data}\left(x\right)$. The proposed network can disentangle the identity-related attributes of the secret or the QR-coded message from the non-identity-related attributes of the cover. Then, we design specific rate-distortion loss functions to control the relationship between the visibility of the cover image and the compressibility of the secret. After that, we analyze the corresponding physical meaning based on information theory, including (a) the mutual information between the cover and the stego-images and (b) the mutual information between the authentic secret and the reconstructed secret images. The detailed specific rate-distortion loss functions will be explained in Section 3.2.1.

**Figure 2:**
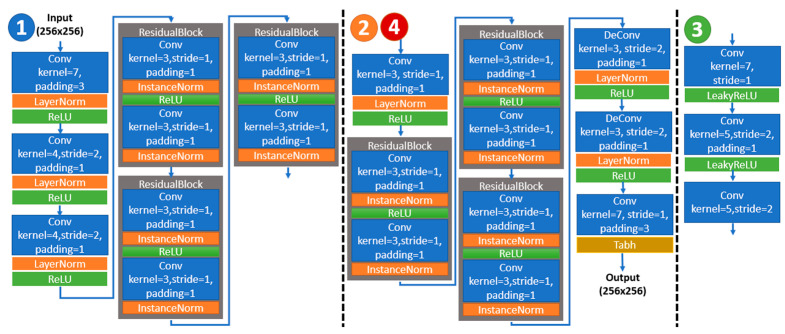
The layer structures of each subnetwork, indicated in Figure 1, for the proposed RD-Stego system.

From the labels in Figure 1 above, we correspond these with those in Figure 2 and show the details about the individual network layers in each component of our proposed RD-Stego architecture. As outlined in Figure 2, to analyze the entire RD-Stego system from the perspective of a network structure, the Feature Extractor is used to downsample and executes the subsequent processing operations for the input three-channel color images. Conversely, the Hiding Network and the Reveal Network perform the upsampling task and rebuild the three-channel color images. Therefore, the basic structures of the Hiding Network and the Reveal Network are the same, but their purposes are different. The purpose of The Hiding Network is to hide the secret-related features and generate the stego-image. In contrast, the primary purpose of the Reveal Network is to process the reconstructed secret images after obtaining the secret-related features. The task of the Latent Encoder is relatively independent, mainly focusing on calculating the loss associated with the rate term.

### 3.2. The Disentangle Efficacy of the Designed Loss Functions

#### 3.2.1. Rate-Distortion Loss Functions

The training goal is to minimize the expected length of the bitstream as well as the expected distortion of the reconstructed stego-image and multiple secret images with respect to their original versions, giving rise to the following rate-distortion optimization problem:

(1) $$ℛ+\lambda *D=\overset{rate}{\overbrace{E_{x\sim P_{x}}\left[-log_{2}p_{\hat{c^{\prime }}}\left(\langle En\left(x\right)\rangle \right)\right]}}+\lambda_{1}*\overset{distortion\left(encoder\right)}{\overbrace{E_{x\sim P_{x}}\left[d\left(c,\langle En\left(x\right)\rangle \right)\right]}}+\lambda_{2}*\overset{distortion\left(decoder\right)}{\overbrace{E_{x\sim P_{x}}\left[d\left(x,\langle De\left(c^{\prime }\right)\rangle \right)\right]}}$$

where λ is the Lagrange multiplier determining the desired rate-distortion trade-off, $P_{x}$ is the unknown distribution of a chosen image x. X is the set of cover images and multiple secret-related images, so that we can define $x\in X=\left\{c,\;s_{1},s_{2},\ldots ,s_{n}\right\}$. Let 〈.〉 denote the rounding to the nearest integer operator (i.e., the quantizer). The stego-image is the output after encoding the chosen image x, and we can define the corresponding stego-image as $c^{\prime }=En\left(x\right)$. Thus, $\hat{c^{\prime }}=\langle c^{\prime }\rangle$ are the quantized latents, $p_{\hat{c^{\prime }}}$ is the discrete probability model associated with $\hat{c^{\prime }}$. $x^{\prime }$ is the output after conducting the decoding process, that is, $x^{\prime }=De\left(c^{\prime }\right)$, so $x^{\prime }$ represents the combined result of the reconstructed cover image and the reconstructed secrets. In Equation (1), the rate term stands for the cross-entropy between the marginal distribution of the latents and the learned entropy model, which will be minimized when the two distributions are identical. The distortion term may correspond to a closed-form likelihood ratio when $d\left(c,c^{\prime }\right)$ and $d\left(x,x^{\prime }\right)$ are measured by the mean squared error (MSE) between their concerning arguments. Under such conditions, the model can be interpreted as a variational autoencoder (VAE). When optimizing the model using other perceptual distortion metrics, such as SSIM or MS-SSIM, the distortion terms can simply be treated as subjective perceptual distance functions to be minimized.

Firstly, to discuss the acquisition of the rate loss function, we calculate the cross-entropy between $c^{\prime }$ and $\hat{c^{\prime }}$, as expressed in Equation (2). The intention is to use the rate loss function to form a compression ratio control factor—${ℒ}_{c^{\prime },\hat{c^{\prime }}}$. This factor controls the rate at which the cover image can be adequately compressed within a visually acceptable range. Moreover, when the compression is complete, the remaining bit budget has to leave enough room to allow for the embedded multiple secret images to coexist. In other words, these multiple secret images also need to go through a certain degree of compression to fit in the original capacity constraints.

(2) $${ℒ}_{c^{\prime },\hat{c^{\prime }}}=E_{x\sim P_{x}}\left[-log_{2}p_{\hat{c^{\prime }}}\left(\langle c^{\prime }\rangle \right)\right],$$

Secondly, let us discuss the encoder distortion loss function. We optimize the weights of the encoder network through adversarial training. Thus, we use the L_1_ smooth loss function (denoted as |. |1; smooth in Equation (3)) to constrain the distance between the cover image (such as c) and the stego-image (such as $c^{\prime }$). The encoder’s distortion loss function can be expressed as:

(3) $${ℒ}_{c,c^{\prime }}=E_{c\sim P_{En}}\left[\frac{1}{3\times W\times H}|c-En\left(s_{1},s_{2},\ldots ,s_{n},c\right){|}_{1;smooth}\right],$$

where the smooth $L_{1}$ loss can be interpreted as a combination of conventional $L_{1}$ loss and $L_{2}$ loss. It behaves as an $L_{1}$ loss when the absolute value of the argument is high (i.e., larger than the given threshold α), and it behaves like an $L_{2}$ loss when the absolute value of the argument is close to zero. Mathematically, we express it as:

(4) $$L_{1;smooth}=\{\begin{array}{c} \left|x\right|\;\;\;\;\;\;\;\;\;if\;\left|x\right|>\alpha \\ \frac{1}{\left|\alpha \right|}x^{2}\;\;\;\;if\;\left|x\right|\leq \;\alpha \end{array}$$

The smooth L_1_ loss combines the advantages of L_1_ loss (steady gradients for large values of *x*) and L_2_ loss (less oscillations during updates when *x* is small).

Finally, let us focus on the distortion loss functions designed for the decoder. The decoder is in charge of reconstructing the secret-related information. We also use the smooth L_1_ loss to measure the similarity between the secret-related images S and the reconstructed secret-related images $S^{\prime }$, where $S^{\prime }≜\;\{{s^{\prime }}_{1},\;{s^{\prime }}_{2},\ldots ,{s^{\prime }}_{n}\}$. The decoder’s distortion loss functions can be expressed as:

(5) $${ℒ}_{s_{1},{s^{\prime }}_{1}}=E_{s_{1}:secret\sim p_{De}}[\frac{1}{3\times W\times H}|\left(\;s_{1}-De\left(c^{\prime }:\in {s^{\prime }}_{1}\right){|}_{1;smooth}\right]$$

and:

(6) $${ℒ}_{s_{2},{s^{\prime }}_{2}}=E_{s_{2}:qrcode\sim p_{De}}[\frac{1}{3\times W\times H}|\left(\;s_{2}-De\left(c^{\prime }:\in {s^{\prime }}_{2}\right){|}_{1;smooth}\right]$$

and:

(7) $${ℒ}_{s_{n},{s^{\prime }}_{n}}=E_{s_{n}:secret\sim p_{De}}[\frac{1}{3\times W\times H}|\left(\;s_{n}-De\left(c^{\prime }:\in {s^{\prime }}_{n}\right){|}_{1;smooth}\right]$$

After defining the rate-distortion loss functions of the encoder and the decoder, we can form the overall adversarial loss function as:

(8) $${ℒ}_{adv}={ℒ}_{c^{\prime },\hat{c^{\prime }}}+\lambda_{c}{ℒ}_{c,c^{\prime }}+\lambda_{s_{1}}L_{s_{1},{s^{\prime }}_{1}}+\lambda_{s_{2}}L_{s_{2},{s^{\prime }}_{2}}+\ldots +\lambda_{s_{n}}L_{s_{n},{s^{\prime }}_{n}}.$$

We use the following parameter settings, $\lambda_{c}$ = 2, $\lambda_{s_{1}}$ = $\lambda_{s_{2}}$ =…= $\lambda_{s_{n}}$ = 1, for conducting all the experiments in this work; we had to consider making the stego-image more visually similar to the cover image and, at the same time, maintain the same clarity of each secret image when dealing with multiple hidden secret images. This requirement also makes us choose the weight of $\lambda_{c}$ to be larger than the weight of $\lambda_{s_{1}}$, $\lambda_{s_{2}}$,…,$\lambda_{s_{n}}$, which are the same weights recommended for each secret image (i.e., $\lambda_{s_{1}}$ = $\lambda_{s_{2}}$ =…= $\lambda_{s_{n}}$). The system will set the weights according to the number of embedded images. For example, if there are two secret images to be embedded, the system will set $\lambda_{c}$ = 2, and $\lambda_{s_{1}}$ = $\lambda_{s_{2}}$ = 1; or $\lambda_{c}$ = 4, $\lambda_{s_{1}}$ = $\lambda_{s_{2}}$ = 2, which means we keep the ratio between $\lambda_{c}$: $\lambda_{s_{i}}$ = 2:1, where i is the number of embedded images. Intuitively, the reconstructed images will be blurred, or the color cast problem will get serious if the number of embedded payload increases. Empirically we found that when we set the ratio of $\lambda_{c}$: $\lambda_{s_{i}}$ to 2:1 or 4:1, our RD-Stego provides acceptable quality of the reconstructed secret images. How to find the best ratio, of course, needs to be invested further, and we mark this as one of our future works.

#### 3.2.2. The Overall Loss Function and the Discriminator

We use the PatchGAN-D [29] as our discriminator, denoted as D in the rest of this writeup. The primary purpose of D is to judge whether the cover and the stego-images, the secret and the reconstructed secret messages, and the QR-coded and the reconstructed QR-coded images are similar. Therefore, we design the following closeness classification loss functions, ${ℒ}_{cls}$, to be in charge of correcting the discriminator in the proposed Stego-system. ${ℒ}_{cls}$ includes the following sub-classification loss functions:

- ${ℒ}_{cls_{c}}≜-E_{X\sim p_{En}}\left[\log D(En(c,s_{1},s_{2},\ldots ,s_{n}|y_{c}\;))\right]$. This loss guarantees D will accurately classify the cover image to the stego-image associated with the label information $y_{c}$ and correct for the bias of the encoder.
- ${ℒ}_{cls_{s_{1}}}≜-E_{X\sim p_{De}}\left[\log D(De(c^{\prime }|y_{s_{1}}\;))\right]$. This loss guarantees D will accurately classify the first secret image to the first reconstructed secret image associated with the first secret label information $y_{s_{1}}$, and correct for the bias of the decoder.
- ${ℒ}_{cls_{s_{2}}}≜-E_{X\sim p_{De}}\left[\log D(De(c^{\prime }|y_{s_{2}}\;))\right]$. This loss guarantees D will accurately classify the second secret image to the second reconstructed secret image associated with the second secret label information $y_{s_{2}}$ and correct for the bias of the decoder.
- ${ℒ}_{cls_{s_{n}}}≜-E_{X\sim p_{De}}\left[\log D(De(c^{\prime }|y_{s_{n}}\;))\right]$. This loss guarantees D will accurately classify the nth secret image to the *n*th reconstructed secret image associated with the *n*th secret label information $y_{s_{n}}$, and correct for the bias of the decoder.

Thus, the overall closeness loss function becomes ${ℒ}_{cls}={ℒ}_{cls_{c}}+{ℒ}_{cls_{s_{1}}}+{ℒ}_{cls_{s_{2}}}+\cdots +{ℒ}_{cls_{s_{n}}}$. Now, taking the adversarial loss function into account, the total embedding loss function would be:

(9) $$ℒ={ℒ}_{adv}+{ℒ}_{cls}.$$

As for the discriminator, the following loss functions are included:

- $D\_{ℒ}_{cls_{c}}≜-E_{X\sim p_{En}}\left[\log D\left(En\left(c,s_{1},s_{2},\ldots ,s_{n}|y_{c}\;\right)\right)\right]-E_{X\sim p_{En}}[\log D(c|y_{c})]$. This loss guarantees that D will accurately correct its bias with the aid of the cover image label information $y_{c}$.
- $D\_{ℒ}_{cls_{s_{1}}}≜-E_{X\sim p_{De}}[\log D\left(De\left(c^{\prime }|y_{s_{1}}\;\right)\right)]-E_{X\sim p_{En}}[\log D(s_{1}|y_{s_{1}}\;)]$. This loss guarantees that D will accurately correct its bias with the aid of the secret label information $y_{s_{1}}$.
- $D\_{ℒ}_{cls_{s_{2}}}≜-E_{X\sim p_{De}}[\log D\left(De\left(c^{\prime }|y_{s_{2}}\;\right)\right)]-E_{X\sim p_{En}}[\log D(s_{2}|y_{s_{2}})]$. This loss guarantees that D will accurately correct its bias with the aid of the second secret image label information $y_{s_{2}}$.
- $D\_{ℒ}_{cls_{s_{n}}}≜-E_{X\sim p_{De}}[\log D\left(De\left(c^{\prime }|y_{s_{n}}\;\right)\right)]-E_{X\sim p_{En}}[\log D(s_{n}|y_{n})]$. This loss guarantees that D will accurately correct its bias with the aid of the *n*th secret image label information $y_{s_{n}}$.

Therefore, the total discriminator loss can be expressed as:

(10) $${ℒ}_{D}=\Upsilon_{c}D\_{ℒ}_{cls_{c}}+\Upsilon_{s_{1}}D\_{ℒ}_{cls_{s_{1}}}+\Upsilon_{s_{2}}D\_{ℒ}_{cls_{s_{2}}}+\cdots +\Upsilon_{s_{n}}D\_{ℒ}_{cls_{s_{n}}}$$

where the settings $\Upsilon_{c}=\Upsilon_{s_{1}}=\Upsilon_{s_{2}}=\cdots =\Upsilon_{s_{n}}=0.5$ are used in this writeup.

### 3.3. The Information-Theoretic Based Analyses—Cost Functions

For stabilizing the trained model, some cost functions are designed to guide the learning of RD-Stego. We consider both our system’s visual acceptability and recovery radiality more specifically.

#### 3.3.1. Visual Acceptability

To provide a certain degree of visual acceptability, we use the following minimax game to regularize the maximal lower bound of the incurred distortion between the reconstructed cover and the stego-images. Our target is to maximize the acceptable perception range related to the cover and stego-images. That is:

(11) $$min_{En}max_{D}V_{I}\left(D,\;En\right)=V\left(D,En\right)-\lambda_{1}I\left(c;En\left(S,\;c\right)\right).$$

**Figure 3:**
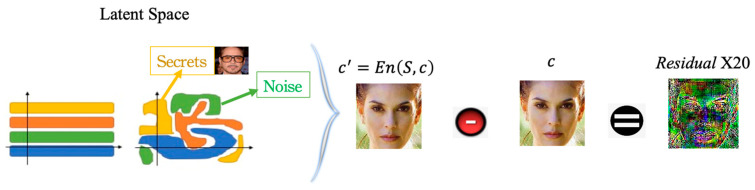
In the latent space, an illustration of the visual similarity between the stego-image and the cover image. This visual closeness is achieved under the constraint of the proposed visual acceptability cost function, which prevents secret-related information from being attacked by sorting out the latent space.

Its primary purpose is to ensure that the stego-image generated by the RD-Stego system can visually approximate the cover image under the control of the visual acceptability cost function. It can also prevent secret-related information from being attacked by sorting out the latent space. The visual acceptability can avoid attackers from making the chosen adaptive cover attack to cause secret-related information omissions, as shown in Figure 3 below.

As sketched in Figure 3, the inputs to the RD-Stego Encoder are the original cover image and the multiple secret images. After completing the encoding, the output will be a latent space representation of the stego-image. This latent space representation contains latent codes associated with the secret-related image features, the cover image features, and noises. Through the designated visual acceptability cost function, the proposed RD-Stego ensures that the latent codes corresponding to essential features of the secret-related information are hard to distinguish from one another and keep the stego-image visually similar to the stego-image simultaneously. When RD-Stego faces the chosen cover image attack, attackers simultaneously know the stego-image c’ and the original cover image c. Let us denote the result of multiplying the magnitudes of the difference between c’ and c by twenty as “Residual × 20”. As evident by the snapshots of Residual × 20, as shown in Figure 3, the RD-Stego leaks nearly no secret-related information.

The relation between the cover image c and the set of secret-related images $S=\{s_{1},\;s_{2},\ldots ,s_{n}\}\;$ can also be represented as $En\left(S,c\right)=c^{\prime }$ after processing through the encoder’s function and then producing a stego-image. Here, the stego-image is denoted as $c^{\prime }$.

In the following, we regularize the objective function of the encoder by maximizing the mutual information between the cover and the stego-images to derive a lower bound for the tolerable visual difference between the cover and the stego-images. Let c represent the latent codes of the cover image, and S $=\{s_{1},\;s_{2},\ldots ,s_{n}\}\;$ be the set of embedded secrets. We treat S as a set of random variables in the following discussions. From the information-theoretic viewpoint, we can use the mutual information (MI), I (X;Y), between the two random variables, *X* and *Y*, to measure the “amount of information” learned for *X* from knowing *Y*, and vice versa. Mathematically, we can represent the MI between *X* and *Y* as:

(12) I(X; Y)=H(X)−H(X|Y)=H(Y)−H(Y|X). 

Therefore, the MI (or the distribution distance) between the cover and the stego-images can be expressed as $I\;\left(c;c^{\prime }\right)=I\left(c;En\left(S,c\right)\right)$. We can derive the maximal value of $I\;\left(c;c^{\prime }\right)$ because a deterministic and invertible encoding function, *En*(.), is used to relate c and $c^{\prime }$. This interpretation makes it easy to formulate a cost function for constraining the visual difference between c and $c^{\prime }$ within a specific range, which is one of the essential requirements in steganography. From the machine learning viewpoint, the above expression stands for the information contained in the latent code of c will not be lost too much in the generation process of the encoder. According to Equation (12), $I\;\left(c;c^{\prime }\right)$ can be expressed as:

(13) $$I\left(c;c^{\prime }\right)=I\left(c;En\left(S,c\right)\right)=H\left(c\right)-H\left(c|En\left(S,c\right)\right)$$

Although, as mentioned above, the encoding function *En*(.), which relates c to $c^{\prime }$ is deterministic and invertible. However, it is hard to directly find the maximal value of Equation (13) because of lacking knowledge about the posterior probability p(c|En(S,c)). We approach this difficulty in computing the mutual information of the encoder by using a variational approximation as follows. Let p(x) denote the distribution of the data x, and we need to bound H(c|En(S,c)) suitably. The positive characteristic of Kullback–Leibler (KL) divergence tells us that:

(14) $$\sum_{c}p(c|En\left(S,c\right))log\;p(c|En\left(S,c\right))-p(c|En\left(S,c\right))log\;q(c|En\left(S,c\right))\geq 0$$

where q(c|En(S,c)) is an arbitrary obtainable variational distribution. Therefore,

(15) $$\begin{array}{cc} I\left(c;En\left(S,c\right)\right)= & H\left(c\right)-H\left(c|En\left(S,c\right)\right)\\ & \geq H\left(c\right)+{\langle \log q\left(c|En\left(S,c\right)\right)\rangle }_{p\left(c,En\left(S,c\right)\right)}\\ & ≜\tilde{I}\left(c;En\left(S,c\right)\right) \end{array}$$

where $H\left(c\right)={-\langle \log p\left(c\right)\rangle }_{p\left(c\right)}$, $H\left(c|En\left(S,c\right)\right)={-\langle \log p\left(c|En\left(S,c\right)\right)\rangle }_{p\left(c,En\left(S,c\right)\right)}$, and $\tilde{I}\left(c;En\left(S,c\right)\right)\;\mathrm{are}\;$ approximations of I(c;En(S,c)) based on q(c|En(S,c)). In other words, the meaning of KL divergence tells us that the relation indicated in Equation (15) is equivalent to depicting a moment matching approximation of p(c|En(S,c)) by q(c|En(S,c)). Let’s view En(S,c) as an information channel with input c and output c’, the probability of constructing $c^{\prime }$ given c can be expressed as:

(16) $$\begin{array}{cc} \log p\;\left(c^{\prime }|c\right) & =\log\int_{En\left(S,c\right)}p\left(c^{\prime }|En\left(S,c\right)\right)p\left(En\left(S,c\right)|c\right)\;\\ & {\geq \langle log\;p\left(c^{\prime }|En\left(S,c\right)\right)\rangle }_{p(En\left(S,c\right)|c)} \end{array}$$

After averaging Equation (16) over all possible c and combining it with the approximation result obtained in Equation (15), we have:

(17) $$\begin{array}{c} \sum_{c}p\left(c\right)\log p\left(c^{\prime }|c\right)\;\geq \;\sum_{c}{\langle \log p\left(c^{\prime }|En\left(S,c\right)\right)\rangle }_{p\left(c,En\left(S,c\right)\right)}\\ \approx {\langle log\;q\left(c|En\left(S,c\right)\right)\rangle }_{p\left(c,En\left(S,c\right)\right)} \end{array}$$

By exchanging the terms on the different sides of Equation (15), we have:

(18) $$H\left(c|En\left(S,c\right)\right)\geq H\left(c\right)-\tilde{I}\left(c;En\left(S,c\right)\right).$$

Equation (18) can be used to derive the lower bound of the prediction error of c by giving En(S,c)  measured based on q(c|En(S,c)). Now, for a fixed p(c), finding the maximization of $\tilde{I}\left(c;En\left(S,c\right)\right)\;\mathrm{measured}\;\mathrm{based}\;\mathrm{on}\;q(c|En\left(S,c\right))$ is equivalent to computing the desired lower bound.

#### 3.3.2. Recovery Fidelity

As for the recovery fidelity, we also use the minimax game to maximize the lower bound of the incurred distortion between the embedded secret and the reconstructed secret images. Our target is to maximize the retrieval fidelity of the embedded messages. Thus, we can write the information-theoretical cost function for designing a practical decoder of our RD-Stego system as:

(19) $$min_{De}max_{D}V_{I}\left(D,De\right)=V\left(D,De\right)-\lambda_{2}I\left(S;De\left(c^{\prime }\right)\right)=V\left(D,De\right)-\lambda_{2}I\left(S;S^{\prime }\right).$$

**Figure 4:**
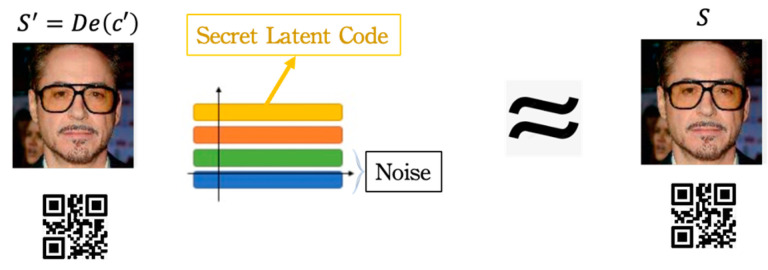
With the proposed fidelity cost function, RD-Stego can maximally restore the embedded secret message from the stego-image and erase the noise.

The primary goal of adopting the recovery fidelity cost function is to maximally restore the original secret message from the contaminated stego-image and erase the incurred noise as much as possible through the operation of the decoder. Figure 4 conceptualizes the effectiveness of the proposed fidelity cost function.

Suppose we view $De\left(c^{\prime }\right)$ as another information processing channel and let $S^{\prime }\;\mathrm{be}\;\mathrm{its}\;\mathrm{output}.$ In that case, the MI between S and $S^{\prime }$, $I\left(S;S^{\prime }\right)\;$ provides an effective tool for measuring the reconstruction quality of the proposed stego system. This is because stego-image $c^{\prime }$ contains the information related to the embedded secret, which is helpful to give the decoder an appropriate guide. That is, we can use the information $De\left(c^{\prime }\right)$ to reconstruct the secret back into $S^{\prime }$. Based on the symmetric property of MI, we can obtain the following equation:

(20) $$I\left(S;S^{\prime }\right)=I\left(S;De\left(c^{\prime }\right)\right)=H\left(S\right)-H\left(S|De\left(c^{\prime }\right)\right).$$

Similar to Section 3.3.1, we want to bound $H\left(S|De\left(c^{\prime }\right)\right)$, and once again, the positivity property of the Kullback–Leibler divergence gives us:

(21) $$\sum_{S}p(S|De\left(c^{\prime }\right))log\;p(S|De\left(c^{\prime }\right))-p(S|De\left(c^{\prime }\right))log\;q(S|De\left(c^{\prime }\right))\geq 0.$$

Therefore,

(22) $$I\left(S;De\left(c^{\prime }\right)\right)=H\left(S\right)-H\left(S|De\left(c^{\prime }\right)\right)\;\geq H\left(S\right)+{\langle \log q\left(S|De\left(c^{\prime }\right)\right)\rangle }_{p\left(S,De\left(c^{\prime }\right)\right)}\;≜\tilde{I}\left(S;De\left(c^{\prime }\right)\right).$$

where $q(S|De\left(c^{\prime }\right))$ is another variational distribution, obtainable at the decoder site. Since our derivation is also based on KL divergence, the relation indicated in Equation (22) is again equivalent to a moment matching approximation of $p\left(S|De\left(c^{\prime }\right)\right)$ by $q\left(S|De\left(c^{\prime }\right)\right)$. Hence, when we fixed p(S), doing the maximization of $\tilde{I}\left(S;De\left(c^{\prime }\right)\right)$ is the same as maximizing the lower bound on the probability of correctly reconstructing the secret-related images. It means that the lower bound becomes tight as $\tilde{I}\left(S;De\left(c^{\prime }\right)\right)=H\left(S\right)$ approaches the actual posterior distribution, and the maximal MI is achieved.

The associated experimental results and related discussions about the effects of the cost functions mentioned above will be given in Section 5.

## 4. Experimental Materials and the Related Benchmarking Methods

To verify our claims and justify the applicability of RD-Stego, we conducted a series of experiments and compared the outcomes with some selected benchmarks. This section summarizes the experimental-related materials and the characteristics of selected benchmarking works.

### 4.1. Experimental Environments and Testing Datasets

Table 2 summarizes the characteristics of our experimental environments, including the hardware specifications and software environment settings. We use the following datasets—FaceScrub [14], CASIA-WebFace [15], and CelebA-HQ/CelebA [16] to train RD-Stego and use ImageNet [17] to investigate cross-domain performance. FaceScrub comprises 106,863 face images of 530 male and female celebrities, including 200 images per person. As such, it is one of the largest publicly available face databases. Due to its having about 200 shots per person, RD-Stego can learn the face attributes more efficiently and be effectively applied to other datasets. Besides using FaceScrub to train our model, we use CASIA-WebFace and CelebA-HQ/CelebA to do the validation tasks. CASIA-WebFace has over 453,453 face images of 10,575 people, while CelebA-HQ/CelebA has over 30,000 face images of 10,177 people. The ImageNet dataset contains 14,197,122 annotated images, 1,034,908 images with bounding box annotations, 1000 synsets with SIFT features, and 1.2 million images with SIFT features from the WordNet hierarchy. ImageNet is the most well-known and widely used benchmark for image classification and object detection.

### 4.2. Evaluation Metrics

We dedicate our experiments to the following perceptual-based image quality metrics: the structural-similarity index measure (SSIM) and peak signal-to-noise ratio (PSNR). SSIM aims to measure the quality of steganographic images in brightness, contrast, and structure. The higher SSIM value means higher similarity between the cover and the stego-images. PSNR evaluates the visual quality of images by calculating the error between the two. The larger the PSNR values, the smaller the distortion between the compared images.

On the other hand, to justify that the behavior of RD-Stego is close to those of the human senses, we also use PieAPP [30], whose primary function is to simulate human perception for quality assessment. Therefore, a lower PieAPP error value is preferred. We also use an existed tool, StegExpose [31], to examine RD-Stego’s anti-steganalysis ability. StegExpose is specialized in detecting LSB steganography in lossless compressed images, such as PNG and BMP processed images.

### 4.3. The Related Benchmarking Methods

Before analyzing the experimental results, we will name several critical NN-based steganography studies, including Deep Steganography [6], Duan et al. [12], SteganoGAN [7], HIGAN [8], and ISN [10]. Noticeably, the methods mentioned above (besides ISN), and the works presented in [6,7,8,12] can only hide a single secret image or text due to the limitation of the restricted model. This study also includes ISN [10], which can conceal multiple private messages, into our performance comparison for completeness. Finally, we will present the detailed analyses of our Experimental Results in the next section.

## 5. Experimental Results and Analysis

To demonstrate the effectiveness of the proposed approach, we conduct both quantitative and qualitative experiments as follows. First, we compare the quantity of RD-Stego with other works. Then, we use the pre-described metrics to evaluate the qualities of the steganographic and the reconstructed images generated by the RD-Stego system. Table 3 reports the subjective (SSIM) and objective (PSNR) quality measurements of the proposed and benchmarked approaches. From Table 3, our RD-Stego produces better qualities in both stego and reconstructed secret images than those produced by existing comparable methods. In the hiding of one image scenario, the performance of the stego-images generated by RD-Stego is better than in previous works regarding SSIM and PSNR values. Although the quality of the reconstructed secret images is not as good as that of Duan et al. [12], the quality is still acceptable and very close to that in [12]. From such experimental results, we can speculate that since the advantage of [12] is to use U-Net to tackle the limited payload capacity, the SSIM and the PSNR values of the reconstructed secret are better. Compared to [12], the advantage of RD-Stego is its ability to increase the payload capacity. In hiding multiple images (e.g., hiding two images), RD-Stego performs better than ISN [10] on both stego-images and reconstructed secret images. This positive result shows that the proposed stego system does make good use of rate-distortion theory for processing multiple hidden messages and ensuring the visibility of the cover and the compressibility of the secret. Therefore, we think RD-Stego could provide a higher embedding capacity than existing approaches. The possible reason is that the multiple secret images and QR-coded features are amplified firstly in the encoding process, and then compression is conducted to increase the amount of transmittable information after doing an elementwise addition and the rate-distortion calculation. Therefore, the embedding capacity that our stego system can handle is more significant than ISN [10]. Since there are three color channels with an 8-bit bandwidth for each, in our experiments, RD-Stego’s embedding payload reaches 192+ bpp. And the limitation of RD-Setgo relies on the physical constraints of the memory space of the GPU accelerator.

**Figure 5:**
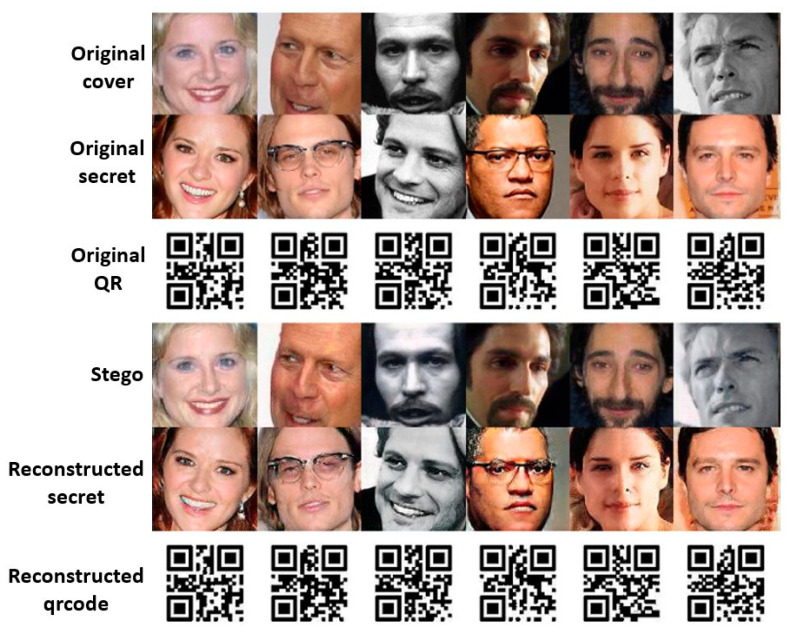
The visual-fidelity investigation of the proposed RD-Stego system: the first row shows the snapshots of the cover images, the second depicts that of the hidden secret photos, the third row presents the snapshots of the hidden QR-coded images, and the fourth is that of the generated stego-images, with the fifth giving the snapshots of the reconstructed secret images and the last row showing the snapshots of the reconstructed QR-coded images.

**Figure 6:**
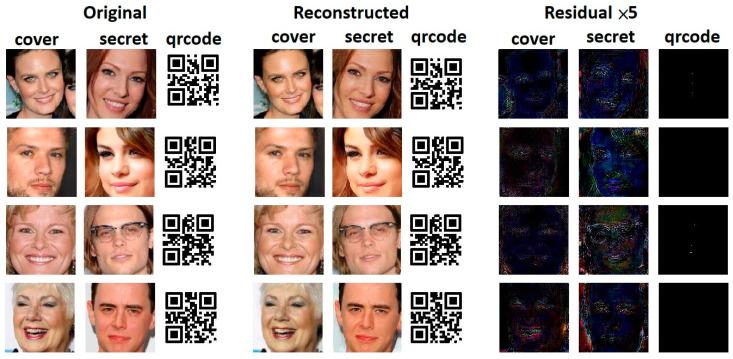
The visual quality investigation of the “Residual × 5”. The leftmost (“Original”) part presents the snapshots of the original cover images, the hidden secret photos, and QR-coded pictures. The Center (“Reconstructed”) part shows the snapshots of the cover images embedded with the secret photos and the QR-coded photos. The proposed RD-Stego system generates the reconstructed secret images and QR-coded images. The rightmost part depicts the magnified residuals obtained from the difference between the cover and the hidden messages. These experimental results evidence that there is nearly no secret-related information leakage during the processes of the proposed stego system.

We now investigate the quality of the generated images (let us take hiding one ordinally image and one QR-coded image as an example). Figure 5 visually presents the snapshots of images generated by our RD-Stego system, including steganographic images and the recovered secret-related information. The quality of those pictures indicates that the proposed method works well in visual fidelity preservation. Suppose attackers have both the cover and the stego-images and launch a chosen cover image attack. We multiply the magnitudes of the difference image (obtained by subtracting the stego-image from the cover image) by five (denoted as “Residual × 5” in the following discussions) and show the results in the rightmost three columns of Figure 6. From the snapshots of “Residual × 5”, it is evident that there is nearly no secret-related information leakage during the processes of the proposed stego system. This positive observation implies that the stego formed by the RD-Stego model provides no signs to attackers for detecting the secret-related information. In other words, our method offers a certain degree of robustness against the chosen cover image attack.

**Figure 7:**
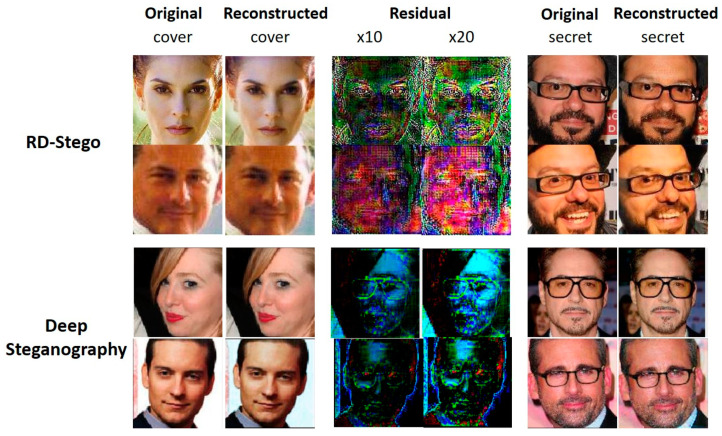
Visual quality investigations for testing cases of “Residual × 10” and “Residual × 20” were conducted based on RD-Stego and Deep Steganography. The top two rows present the results generated using the RD-Stego system, while the bottom two rows depict that of Deep Steganography. We can find some secret-related information (such as the glass-wearing) in the residual images produced by Deep Steganography.

In the tests of “Residual × 10” and “Residual × 20”, depicted in Figure 7, we compare the visual appearances of the related snapshots obtained using the RD-Stego with those of Deep Steganography. The magnified residues evidence that the proposed system provides better security than Deep Steganography [6] since we can detect much less secret-related information from them. Compared to Deep Steganography, the advantages of RD-Stego come from its increased payload capacity and resistance to the chosen cover attack.

**Figure 8:**
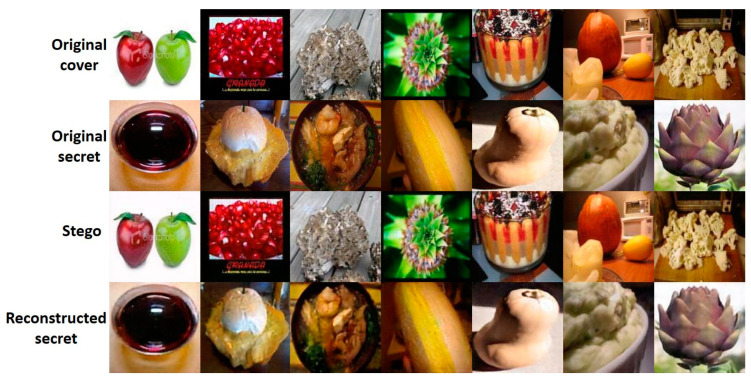
The cross-domain performance testing. Using ImageNet as the testing target, we find only a negligible color cast between the “cover vs. stego” images and the “embedded secret vs. reconstructed secret” messages. In other words, there is almost no high-frequency information loss in the proposed RD-Stego system.

In the next part, we conduct cross-domain verification experiments based on the popular dataset, ImageNet [17]. Notice that the usage of the RD-Stego system is not limited to human faces. According to the snapshots presented in Figure 8, there is nearly no color cast in between the cover vs. stego-images and the secret vs. reconstructed messages. In other words, there is almost no high-frequency information loss in the proposed system. Moreover, our experiments are carried out simultaneously with no cover and secret images appearing in the training dataset.

**Figure 9:**
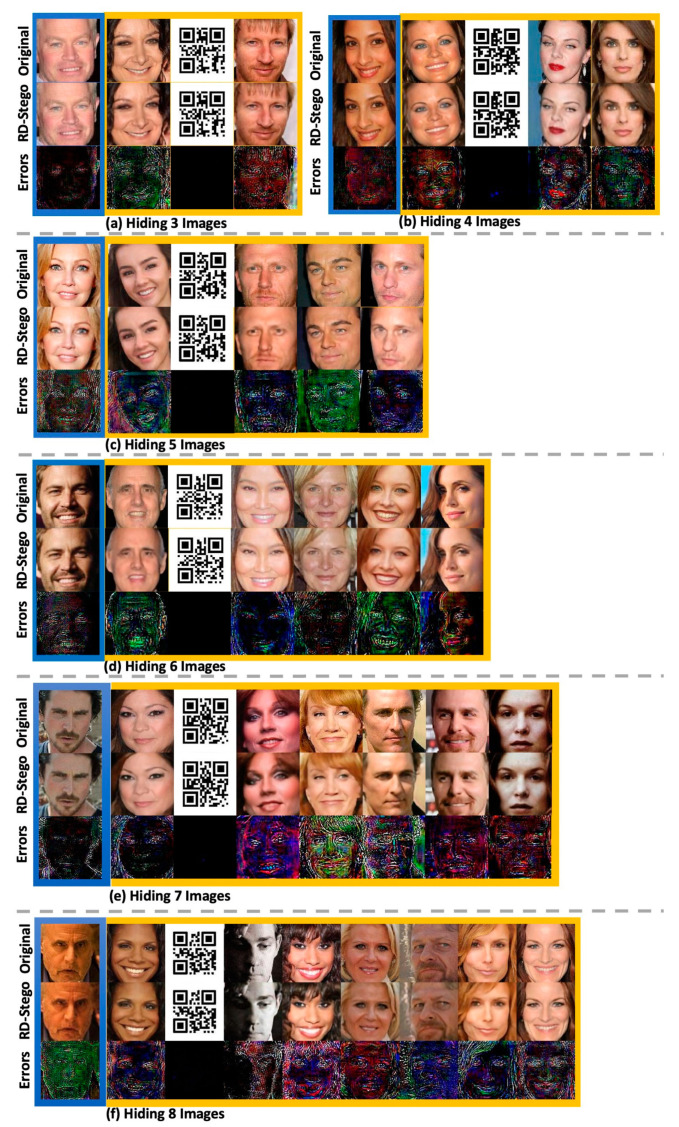
Qualitative investigative results—RD-Stego generated the hiding images up to 192 bpp (i.e., embedded with 3, 4, 5, 6, 7, and 8 images from (**a**) to (**f**)). The residual images are computed as “Residual × 20”.

The following experiment shows one of the strengths of the RD-Stego system—hiding multiple secret images. Benefiting from rate-distortion theory, RD-Stego allows us to hide up to eight color-secret pictures. This limitation comes from the constraint on the simulation platform’s computing resources and the tolerable degree of visual degradation. To justify this claim, we conducted an extra experiment concerning the relation between the RD-Stego’s time spent and GPU memory consumption. The corresponding experimental results are presented in Appendix A. As can be seen from Figure 9, the RD-Stego can handle high-payload secret-related information. Still, the trade-off status is that when a higher amount of data is hidden, the compression rate gets higher, and the larger the high-frequency part of the information that is lost, the worse the color cast problem becomes.

**Figure 10:**
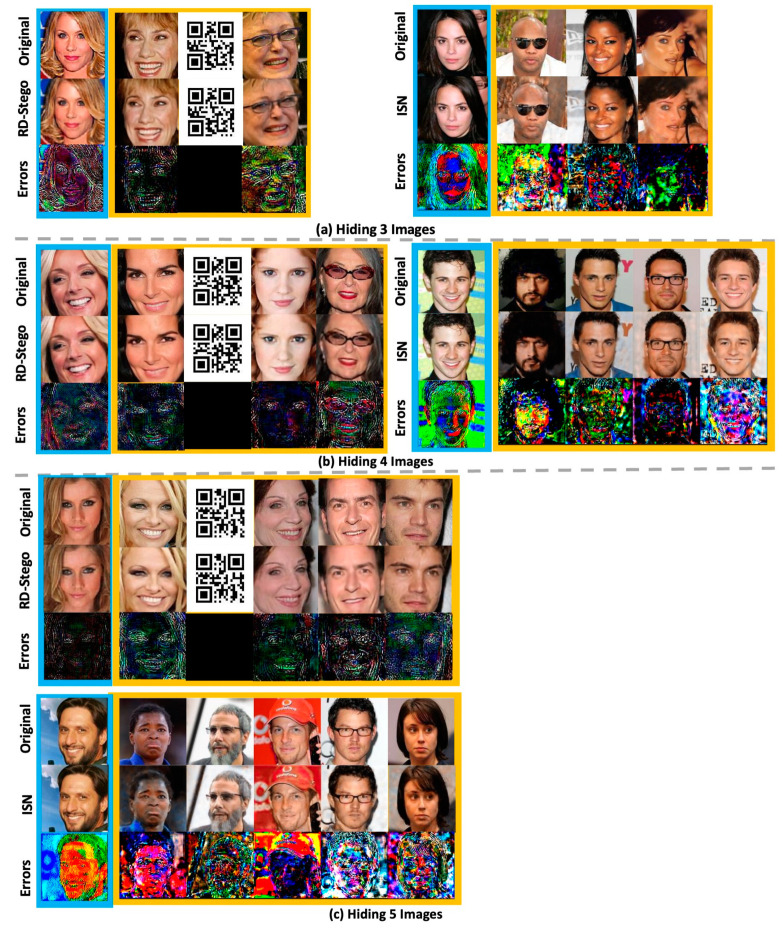
The qualitative examinations of the embedded-information-leakage phenomena, comparing RD-Stego with ISN [10].

We also compare the proposed RD-Stego with the ISN [10], which can hide multiple color images. From Figure 10, we see that ISN can hide multiple secret images very well; however, there is an obvious problem of hidden information leakage. This shortage can be observed by examining the case of hiding four images. The bottom part of Figure 10b shows the original cover images, the stego-images generated by ISN, and the corresponding magnified error images. From the snapshots of the error images, evident information-leakage traces can be found, especially apparent in the ‘wearing glasses’ image (one of the embedded secret images). The above-mentioned information-leakage phenomena can be found in nearly every magnified error image produced by ISN (cf. the bottommost row of Figure 10). Compared with RD-Stego, which can successfully avoid the chosen cover attack, there is no such problem (cf. the left part of Figure 10b). Thus, compared with ISN, the advantages of RD-Stego are its ability to increase the payload capacity and the resistance to the chosen cover attack.

**Figure 11:**
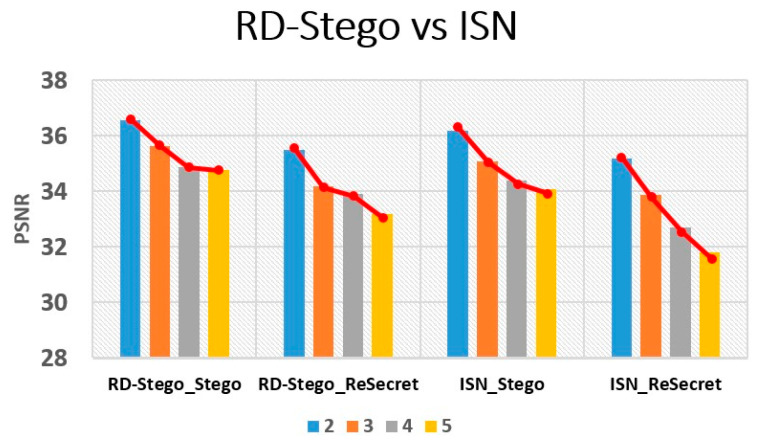
PSNR performance comparison between RD-Stego and ISN [10], when the number of hidden images increases from 2 to 5.

Besides the above information leakage issue, we now empirically analyze the performance drop of RD-Stego caused by the increasing number of hidden images, where ISN is again chosen as our benchmark. Figure 11 shows the PSNR performance drops associated with the stego-images and the reconstructed secret (Reconstructed) images by hiding two, three, four, and five secret images generated by RD-Stego and ISN, respectively. Figure 11 shows, indeed, that there are PSNR drops for all tested cases when the number of embedded images increases. Notably, the INS’s PSNR drop in “Reconstructed” is more severe than in RD-Stego because of INS’s information leakage issue, as mentioned above. Moreover, the stego-images’ PSNR performances for both RD-Stego and ISN dropped as the number of embedded images increased. By checking the first and the third chunks of Figure 11, we found that the slope of the PSNR-dropping curve associated with RD-Stego is more even than that of ISN. This fact implies that as the number of embedded secret images grows continuously, ISN’s PSNR drop will worsen more severely. In other words, the higher degree of limitation in RD-Stego’s distortion comes from the effect of the visual acceptability-related cost function. Similarly, RD-Stego’s better performance in reconstructed secret images, we think, is due to the regulation induced by the recovery fidelity-related cost function, which contributes a lot to this issue.

**Figure 12:**
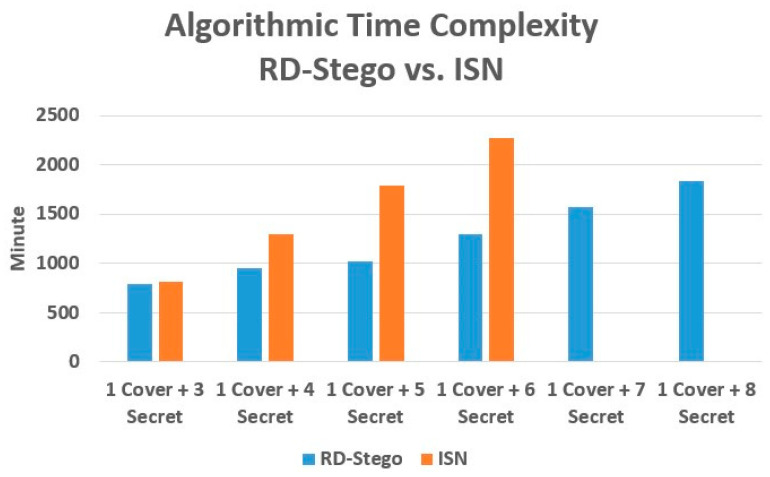
Timing complexity comparison: RD-Stego vs. ISN. The footnote “1 cover + i Secret” in the above figure stands for one cover image embedded with i secret images.

Regarding time complexity, the clever incorporation of the rate-distortion loss function into the design of RD-Stego’s architecture benefits its realization efficiency. With the aid of the loss function mentioned above, we can now use stacks vertically (i.e., we can perform elementwise additions in parallel) to train the encoder, even if multiple secret images are to be embedded simultaneously. This computational structure is very different from that of other benchmarked works. Let us take the state-of-the-art ISN [10] as an example, in which the secret images are concatenated horizontally. This series-natured computing structure will increase the ISN encoder’s computation during the training when the number of hidden images increases. Figure 12 shows the timing performance comparison between the state-of-the-art ISN and the proposed RD-Stego when embedding different numbers of secret images.

As shown in Figure 12, when processing three to six hidden secret images, RD-Stego outperforms ISN in computing time. Moreover, even if RD-Stego is used to hide seven or eight secret images, the required computing time is much less than that of ISN for hiding only six secret images. (We found from our implementation that ISN cannot handle the task of hiding more than six secret images.) To dive into the comparison in a bit more detail, in encoding, after the Feature Extractor performs elementwise additions, the RD-Stego’s Hiding Network will not increase processing time even if a new secret image is added. Similarly, in decoding, the RD-Stego’s Reveal Network will not increase training time when extra hidden images are considered. Therefore, RD-Stego makes hiding multiple secret images easier and needs shorter encoding, decoding, and overall training times than the state-of-the-art ISN.

We also examine the SSIM and PSNR performances of RD-Stego on multiple datasets, as presented in Table 4. Table 4 indicates that RD-Stego performs well on the Celeba and the FaceScrub classes of the ImageNet datasets. Of course, as shown in the last two (ImageNet) columns, performance degradation in both SSIM and PSNR can be expected due to more complicated and variational images without relevant classifications.

**Figure 13:**
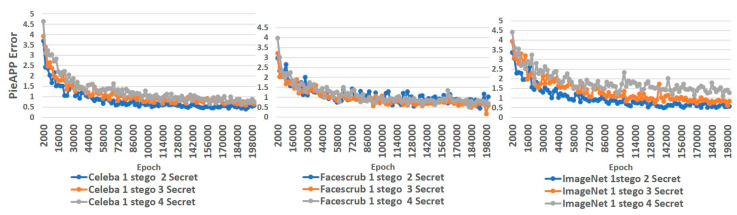
From left to right, the training statuses generated via the application of the perceptual image-error assessment tool PieAPP [30] to Celeba, Facescrub, and ImageNet datasets, respectively.

In the following, two useful and well-developed tools, PieAPP and StegExpose, are applied to justify RD-Stego’s applicability further. PieAPP [30] is a learning-based perceptual image-error assessment tool. We use PieAPP to assess the perceptual errors generated in each epoch during RD-Stego’s training upon different datasets. Figure 13 shows that the error value associated with PieAPP decreases steadily along with epoch evolution. Specifically, all tested cases in CelebA and FaceScrub show the same error evolution trend: the more the secret images are hidden, the closer the error values approach a fixed value of 0.5. This fact indicates that the designated visual acceptability-related cost function is helpful for the convergence of the training process. In contrast, in the ImageNet dataset, the error value is slightly more prominent when the number of hidden images increases; fortunately, the corresponding visual effect is still acceptable for steganographic applications.

We also utilize PieAPP to now analyze the error value of different datasets. For example, according to Table 5, the PieAPP error value of the stego-image generated by the RD-Stego system is outstanding. Relatively, although the error value of the reconstructed secret image is higher than that of the stego-image, its performance is also quite good due to the impact of the recovery fidelity cost function.

**Figure 14:**
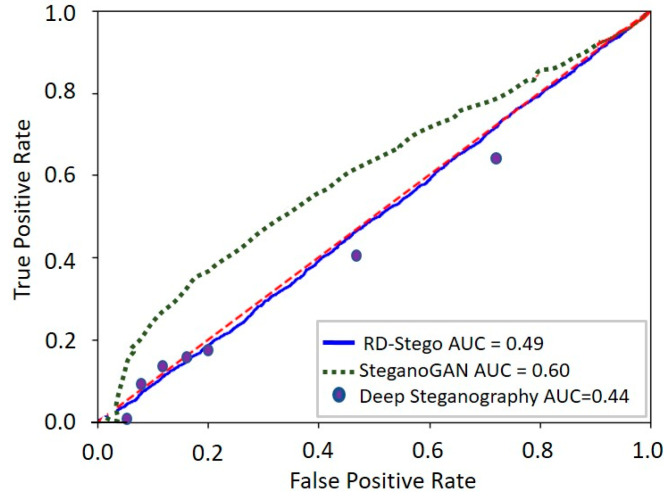
The receiver operating characteristic (ROC)-curves for the proposed RD-Stego, SteganoGAN, and Deep Steganography systems, obtained under the investigation of StegExpose.

Anti-steganalysis ability is an essential characteristic of a good stego system. In response to this challenge, we use an existing tool, StegExpose [31], to examine the RD-Stego’s anti-steganalysis ability. StegExpose is specialized in detecting LSB (least significant bit) steganography in lossless images, such as PNG and BMP. A best-performed stego system should report a detection value of 0.5 upon checking via effective steganalysis tools. This fact means that the tested stego-images can successfully survive being checked through a steganalysis tool, such as the StegExpose. Figure 14 depicts the associated receiver operating characteristic (ROC)-curve of our RD-Stego system. We note that StegExpose is more effective than random guessing in steganalysis, with an area under the ROC curve of 0.49 (very nearly 0.5), even for up to 32-bit payloads. Our method performs better than SteganoGAN (its area under the ROC curve is 0.6) and Baljua’s Deep Steganography (its area under the ROC curve is 0.44). In conclusion, RD-Stego can successfully evade standard steganalysis tools and meet the minimum viable steganography algorithm requirements.

**Figure 15:**
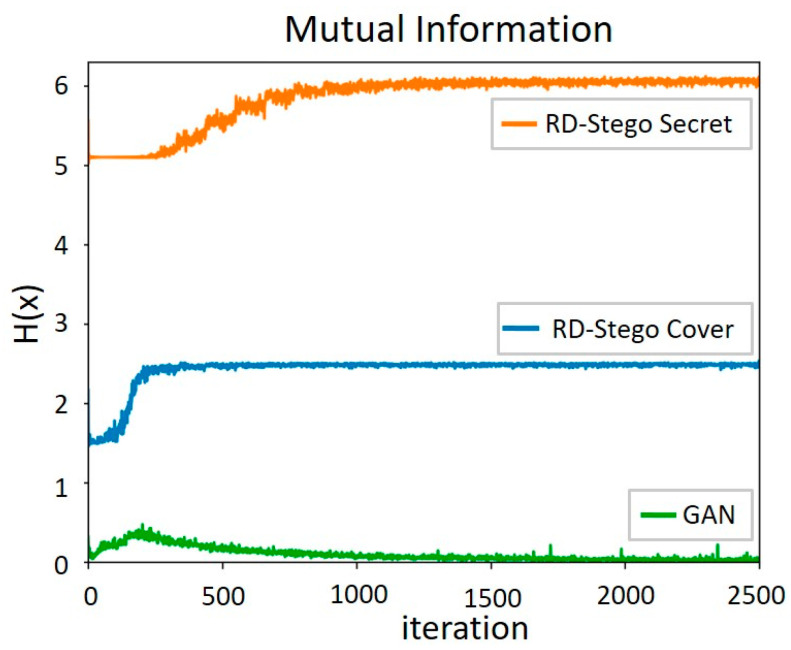
The computed mutual information (MI) $\tilde{I}\left(c;En\left(S,c\right)\right)$ and $\tilde{I}\left(S;De\left(c^{\prime }\right)\right)$ for both the proposed model and a standard GAN model.

Finally, we also conducted the following experiments to justify the effectiveness of the proposed MI-based cost functions. As shown in Figure 15, the encoder’s lower bound $\tilde{I}\left(c;En\left(S,c\right)\right)$ quickly reaches its theoretical maximum H(c)≈ 2.5 bits. This phenomenon means the proposed method can better approach the desired maximal mutual information between the cover and the stego-images than a standard GAN model. Also, this observation demonstrates that our RD-Stego uses latent codes better than a normal GAN. The decoder’s lower bound $\tilde{I}\left(S;De\left(c^{\prime }\right)\right)=H\left(S\right)$ quickly reaches the theoretical maximum H(S)≈ 6 bits. The same statements also hold for the case between the embedding and the reconstructed secret message.

## 6. Conclusions and Future Work

This work proposes a novel rate-distortion-based large-capacity secure semi-supervised cover synthesis steganographic system. To emphasize its foundational origin from Shannon’s information theory, we denote it as the Rate-distortion-based Stego (RD-Stego) system. Our RD-Stego can effectively hide multiple three-channel color images and QR-coded images simultaneously. It can achieve an embedding capacity up to 192 bpp, which is higher than that of existing competing methods. Meanwhile, the proposed stego system provides higher visual fidelity in-between both the cover vs. stego-images and the embedded vs. the reconstructed messages. Furthermore, according to our experiments, the RD-Stego model can resist chosen cover attacks, even if an attacker simultaneously possesses both the stego and the cover images. The superior performances of the proposed work come from newly proposed MI-based cost functions and the rate-distortion theory. Discussions about the mathematical derivation and the physical meaning explanation are also provided for enunciating our design insights. Moreover, our claimed system advantages have been justified by experiments with publicly available datasets.

The proposed RD-Stego is designed and implemented based on rate-distortion theory, which is the leading scientific contribution of this write-up. As a result, RD-Stego dramatically improves the payload capacity in steganography and avoids doubts about the chosen cover attack based on network architecture. Thus, the proposed stego system has guaranteed security. In addition, our current design focuses on stably enlarging the payload capacity with the aid of rate-distortion-based loss functions. Nevertheless, for an ideal secure steganographic system to exist, a certain amount of new information theory-based loss functions should be derived for RD-Stego to face the challenges of various attacks besides the chosen cover one. For example, we should expand the system’s robustness to resist cut-and-paste, compression, noise-adding, and occlusion attacks on the stego-images in the future. In response to this valuable suggestion, we present some preliminary experiments about the performances of RD-Stego against some typical attacks in Appendix B. Finally, increasing RD-Stego’s ability to withstand more complicated steganalysis than the LSB attack is of high interest.

## Figures and Tables

**Table 1:** Comparisons of the advantages and limitations of the proposed RD-Stego and the above-mentioned related works.

Methodology	Payload Capacity	Advantages	Info. Theoretic Based Analyses	Limitations
Deep Stegano. [6], 2017.	Larger than 0.4 bpp	- It is the first process that attempts to address the application of GAN to image steganography with acceptable performance.	-N/A	- The payload capacity needs to be increased. - Poorly performed under the chosen cover attack (CCA).
Duant et al. [12], 2019	8 bpp	- The method is based on a U-Net structure, and the quality of images processed by the method is relatively superior.	-N/A	- The payload capacity needs to be increased. - Poorly performed under CCA and LSB attacks.
SteganoGAN [7], 2019.	4.4 bpp	- The method targets the hiding of arbitrary binary data in an image.	-N/A	- The method is suitable for hiding binary data only. - Poorly performed under the LSB cover attack.
HIGAN [8], 2020.	24 bpp	- HIGAN is the first process that can handle the embedding of one three-channel color image.	-N/A	- The payload capacity needs to be increased. - Poorly performed under CCA and LSB attacks. - The color-cast problem worsens the reconstructed secret images when the number of embedding messages increases.
SteganoCNN [13], 2020.	48 bpp	- SteganoCNN can handle two color images.	-N/A	- The payload capacity needs to be increased. - The color-cast problem worsens the reconstructed secret images when the number of embedding messages increases. - Poorly performed under CCA and LSB attacks.
ISN [10], 2021	24∼120 bpp	- ISN successfully increases the embedding payload capacity to 24∼120 bpp.	-N/A	- Computational time is too long. - Hard to extend the payload capacity. - Poorly performed under CCA.
RD-Stego	192 + bpp	- It is the first write-up engaging the rate-distortion theory in the entire NN architecture for enlarging the hiding capability. - It can resist the LSB attack. - It can resist the chosen cover attack(CCA). - It applies to cross-domain applications.	-Yes	- Performance is dominated by the physical limitations of the GPU accelerator’s memory. - Besides CCA and LSB, the scheme’s robustness to other attacks, such as compression attacks and noise-adding attacks, must be investigated further.

**Table 2:** The hardware specifications and the software environments we used to conduct our experiments.

CPU Model	CPU Memory	Frequency	# of CPU Cores	GPU Model	# of GPU
Intel(R) Xeon(R) Gold 6128 CPU	192 GB	3.4 GHz	24	Tesla V100	2
Operation System	Docker	# of GPUs in Docker	GPU Memory in Docker	CUDA Version	Language
Ubuntu 20.04	20.10.13	1	12GB	11.4	Python 3.7.10 Pytorch 1.9.0

**Table 3:** This table compares the qualities of the steganographic and reconstructed secret images for the proposed and the benchmarked stego systems in SSIM and PSNR.

Method	Hiding Images	Stego (SSIM)	Stego (PSNR)	Re-Constructed Secret (SSIM)	Re-Constructed Secret PNSR)
DeepStegano. [6]	1	0.92	28.41	0.92	28.06
Duan [12]	1	0.95	36.71	0.96	36.97
HIGAN [8]	1	0.94	30.95	0.94	29.67
Ours	1	0.965	36.8	0.94	36.81
ISN [10]	2	0.94	36.2	0.92	35.2
Ours	2	0.96	36.58	0.94	35.5

**Table 4:** The SSIM and PSNR performances of RD-Stego on multiple datasets.

Dataset	CelebA		FaceScrub		ImageNet	
	PSNR/SSIM		PSNR/SSIM		PSNR/SSIM	
	Stego	Secret	Stego	Secret	Stego	Secret
2 Secret	36.58/0.960	35.50/0.931	36.27/0.952	34.86/0.925	34.80/0.932	32.87/0.917
3 Secret	35.64/0.951	34.19/0.925	35.04/0.941	34.05/0.921	34.30/0.923	31.65/0.907
4 Secret	34.86/0.939	33.905/0.913	34.25/0.923	33.75/0.911	33.98/0.914	30.52/0.898
5 Secret	34.76/0.921	33.176/0.906	34.1/0.916	32.15/0.905	33.39/0.901	29.92/0.891
6 Secret	34.5/0.909	31.905/0.901	33.92/0.902	31.02/0.891	32.18/0.896	28.87/0.885

**Table 5:** Analyzed error values for PieAPP on different datasets.

Dataset	CelebA		FaceScrub		ImageNet	
	PieAPP		PieAPP		PieAPP	
	Stego	Secret	Stego	Secret	Stego	Secret
2 Secret	0.110	0.396	0.133	0.385	0.262	0.447
3 Secret	0.131	0.329	0.152	0.392	0.265	0.597
4 Secret	0.169	0.387	0.157	0.450	0.230	0.618
5 Secret	0.163	0.419	0.162	0.475	0.322	0.621
6 Secret	0.215	0.481	0.138	0.562	0.421	0.751

**Figure A1:**
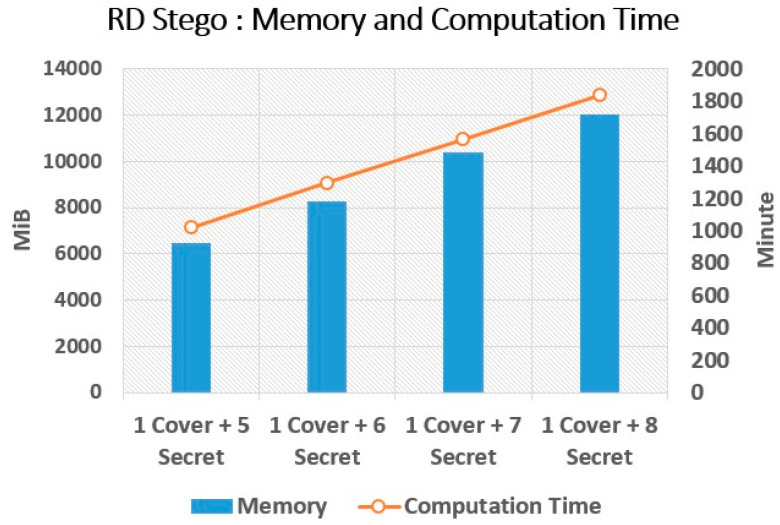
The memory consumption and the computing time of the proposed RD-Stego concerning the different number of embedded images.

**Figure A2:**
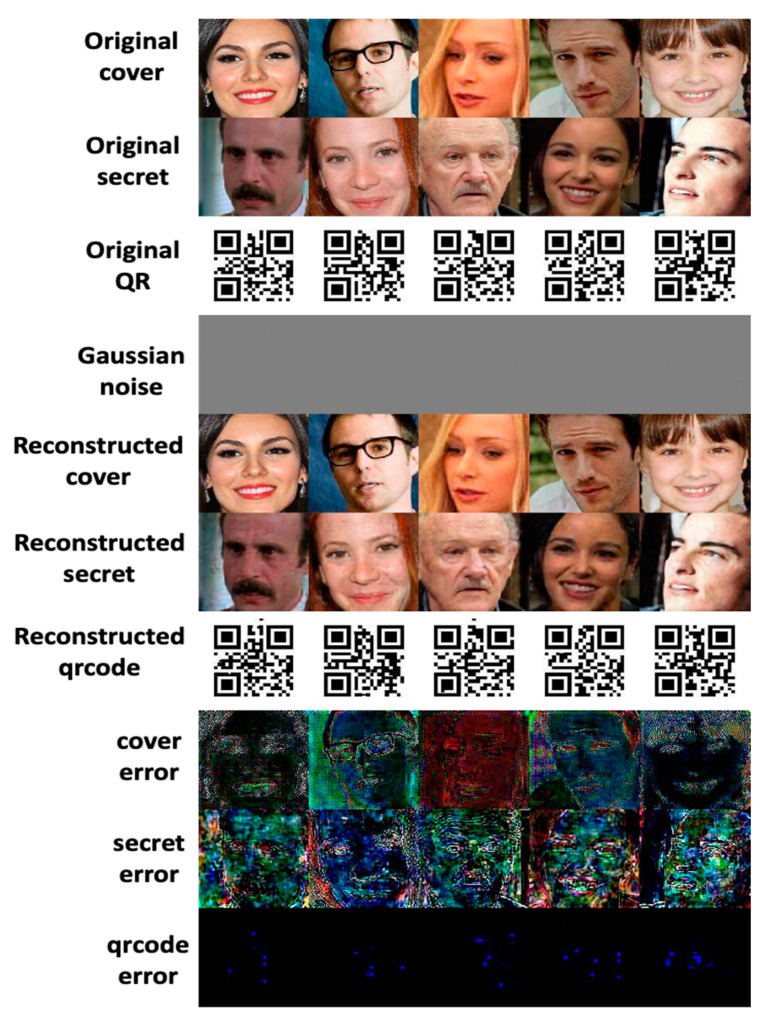
Gaussian noise attack—the RD-Stego system can still successfully reconstruct the facial image and the readable QR-code image.

**Figure A3:**
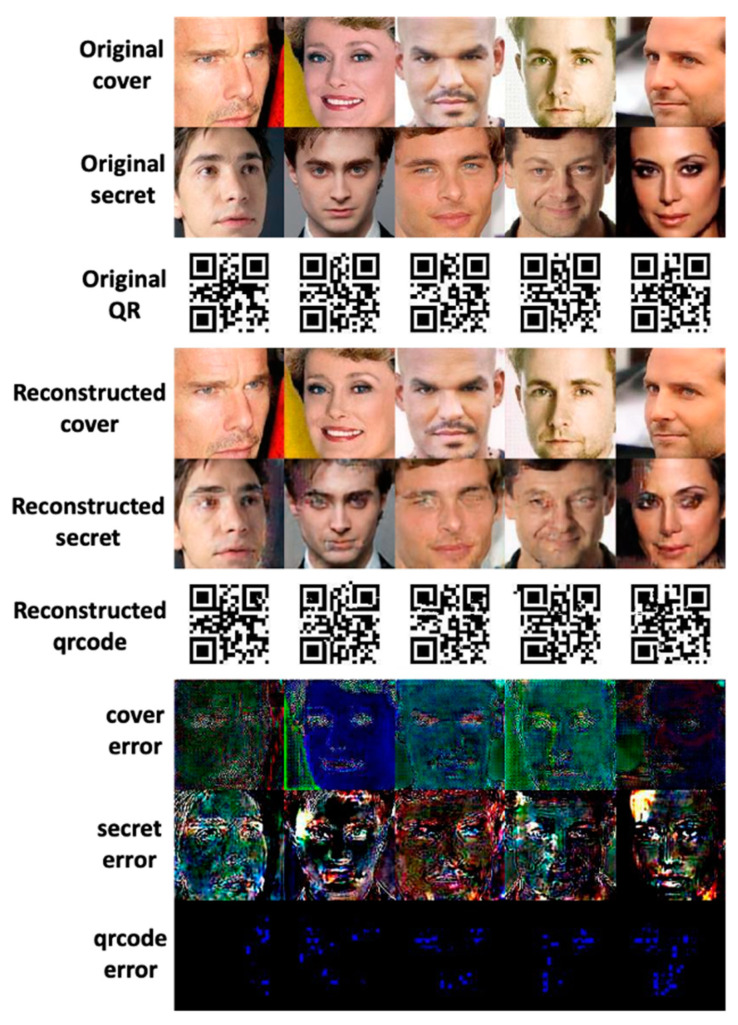
RD-Stego’s performance when it is JPEG-compressed attacked, with a quality factor of 100.

**Figure A4:**
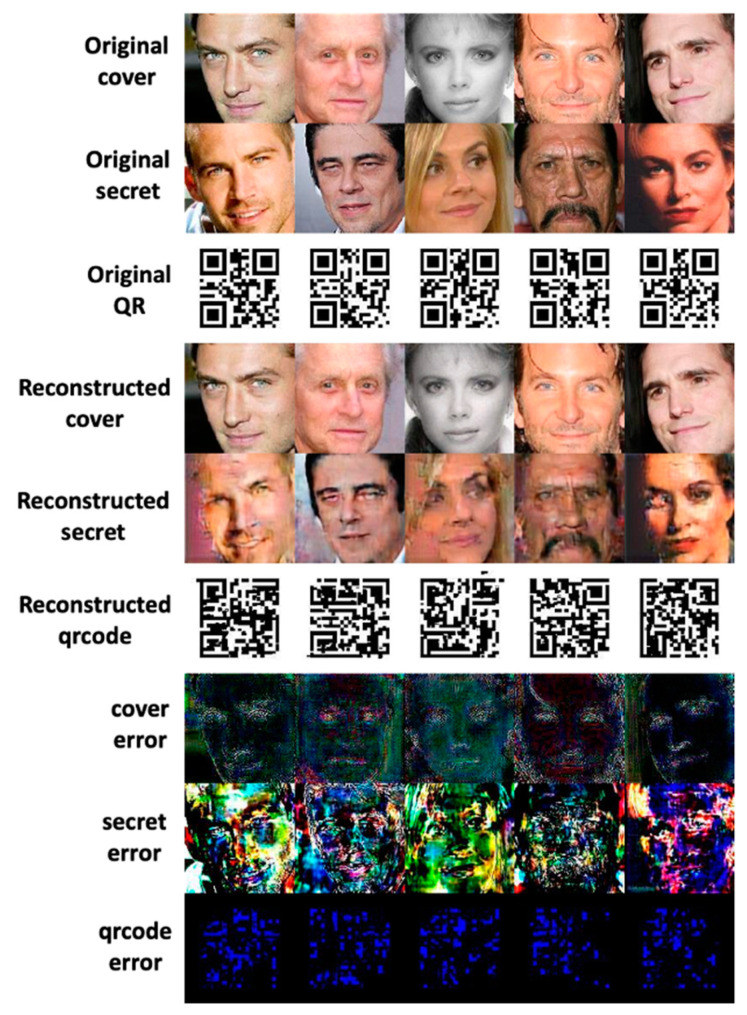
RD-Stego’s performance when it is JPEG-compressed attacked, with a quality factor of 95.

## Appendix A. The Computing Power Limitation of the Proposed RD-Stego

The proposed RD-Stego benefits from rate-distortion theory, allowing us to hide eight color-secret images. In this appendix, we explore the computing (training) time and the GPU memory consumption required by RD-Stego for embedding five, six, seven, and eight secret images. The GPU model used in this experiment is Tesla V100 with 12 GB GPU memory. Figure A1 shows that the GPU memory usage increases by nearly 2000 MiB (2 GiB) from the hiding of five to six secret images. The same situation follows, where we have to consume almost 2000 MiB (2 GiB) extra GPU memory if the number of secret images increases from six to seven. When the number of hidden images reaches eight, the GPU Memory-usage measured by the Nvidia-smi tool is as high as 12,039 MiB (12 GiB), which is almost the physical limitation of the Tesla V100 accelerator. Intuitively, we can deduce that the proposed RD-Stego is capable of hiding more than nine secret images if we run the system on a GPU accelerator with a larger memory capacity. However, as noted earlier, when the number of hidden payloads increases, the compression rate increases, and more high-frequency information will be lost and the color cast problem worsens. Thus, considering the trade-off mentioned earlier, we choose eight as our best number for embedding secret images. Moreover, as shown in Figure A1, if the number of embedded images is increased by 1, the computation time will increase by about 280 min. Thus, the total computation time is as high as 1837 min (approximately 30.6 h) when we embed eight secret images.

## Appendix B. The Performances of RD-Stego under Some Preliminary Attacks

a. Gaussian Noise Attack

When the stego-image is under Gaussian noise attack (assume mean is zero and variance σ is 0.01), as shown in Figure A2, RD-Stego will reconstruct the embedded facial image and the readable QR-code image successfully. Intuitively, we deduce that the higher the number of hidden secret images, the harder the RD-Stego is to resist the Gaussian attack. Of course, it is necessary to design new loss functions for RD-Stego to defend against more complicated and destructive attacks.

b. JPEG Compression Attack

In this experiment, we take the compression attack as an example to examine the responses of RD-Stego. Figure A3 and Figure A4 illustrate the experimental results. We assume the stego-image is under JPEG-compression attack. We use the quality factors 100 and 95 to test the proposed RD-Stego system.

Figure A3 and Figure A4 show that when the stego-image is JPEG compressed, the RD-Stego system can handle only limited payload embedding. This fact justifies again that new information theory-based loss functions are necessary and worthy of development if our design target is to enhance the RD-Stego’s robustness against compression attacks.
